# A Detailed Clinical Case of Localized Prostate Tumors Treated with Nanoparticle-Assisted Sub-Ablative Laser Ablation

**DOI:** 10.3390/nano14151261

**Published:** 2024-07-28

**Authors:** Yara Kadria-Vili, Jon A. Schwartz, Thomas J. Polascik, Glenn P. Goodrich, David Jorden, Diane Pinder, Naomi J. Halas, Ardeshir R. Rastinehad

**Affiliations:** 1 Nanospectra Biosciences Inc., Houston, TX 77054, USA; ykadria-vili@nanospectra.com (Y.K.-V.); david.jorden@nanospectra.com (D.J.); 2 Department of Urology, Duke University Medical Center, Durham, NC 27710, USA; thomas.polascik@duke.edu (T.J.P.);; 3 Laboratory for Nanophotonics, Rice University, 6100 Main Street, Houston, TX 77005, USA; 4 Department of Chemistry, Rice University, 6100 Main Street, Houston, TX 77005, USA; 5 Department of Electrical and Computer Engineering, Rice University, 6100 Main Street, Houston, TX 77005, USA; 6 Department of Physics and Astronomy, Rice University, 6100 Main Street, Houston, TX 77005, USA; halas@rice.edu; 7 Smith Institute for Urology at Lenox Hill Hospital, Northwell Health, Zucker School of Medicine at Hofstra/Northwell, New York, NY 10075, USA

**Keywords:** AuroLase^®^ Therapy, AuroShell^®^ Particles, nanoshells, Ktrans, ADC, dynamic contrast enhancement, multiparametric MRI, prostate cancer, focal ablation, clinical trial

## Abstract

AuroLase^®^ Therapy—a nanoparticle-enabled focal therapy—has the potential to safely and effectively treat localized prostate cancer (PCa), preserving baseline functionality. This article presents a detailed case of localized PCa treated with AuroLase, providing insight on expectations from the diagnosis of PCa to one year post-treatment. AuroLase Therapy is a two-day treatment consisting of a systemic infusion of gold nanoshells (~150-nm hydrodynamic diameter) on Day 1, and sub-ablative laser treatment on Day 2. Multiparametric MRI (mpMRI) was used for tumor visualization, treatment planning, and therapy response assessment. The PCa was targeted with a MR/Ultrasound-fusion (MR/US) transperineal approach. Successful treatment was confirmed at 6 and 12 months post-treatment by the absence of disease in MR/US targeted biopsies. On the mpMRI, confined void space was evident, an indication of necrotic tissues encompassing the treated lesion, which was completely resolved at 12 months, forming a band-like scar with no evidence of recurrent tumor. The patient’s urinary and sexual functions were unchanged. During the one-year follow-up, changes on the DCE sequence and in the Ktrans and ADC values assist in qualitatively and quantitatively evaluating tissue changes. The results highlight the potential of gold-nanoparticle-enabled sub-ablative laser treatment to target and control localized PCa, maintain quality of life, and preserve baseline functionality.

## 1. Introduction

Prostate cancer (PCa) is the second leading cancer-related death in men after lung cancer. The American Cancer Society estimates 299,010 new PCa cases to be diagnosed in 2024 in the United States, leading to 35,250 deaths [1]. Most PCa cases at diagnosis (~80%) are localized and confined within the prostate gland. Early detection and treatment of these low- to intermediate-risk PCas give patients a chance of 99% 10-year relative survival [2]. Whole gland removal with radical prostatectomy can effectively remove localized low- to intermediate-risk cancer. Many PCa survivors treated with radical prostatectomy or whole gland radiotherapy have reported a reduced quality of life due to urinary leakage and/or erectile dysfunction [3,4,5,6] or disturbance in intestinal function [7]. Thus, there is an increased interest in targeted therapies, including focal PCa ablation, for their potential to maintain a good quality of life by preventing damage to the external urethral sphincter and/or neurovascular bundles [8]. Focal therapies include cryoablation [9,10], high-intensity focused ultrasound (HIFU) [11,12], transurethral ultrasound ablation (TULSA) [13], brachytherapy [14], nanoparticle-assisted thermal ablation [15,16], irreversible electroporation (IRE) [17,18,19], vascular-targeted photodynamic therapy (VTP) [20,21], laser interstitial thermal therapy (LITT) [22], and magnetic hyperthermia [23]. All of these therapies are evolving concurrently.

Here, we report on AuroLase^®^ Therapy, a nanoparticle-directed, laser-activated “ultra-focal” treatment of low- to intermediate-risk PCa. This technology involves a systemic infusion of silica/gold (core/shell) nanoparticles, known as “nanoshells” or “AuroShell^®^ Particles”. The United States Food and Drug Administration (FDA) classifies AuroShell^®^ Particles as a device that can selectively ablate soft tissue under near-infrared (NIR) illumination rather than as a drug due to their simple architecture that does not involve the attachment of molecular targeting, fluorescent/radioactive ligands, metabolites, etc. The particles are coated with a 6k-methoxypolyethylene glycol thiol (mPEG thiol), bypassing the macrophage immune system and leading to longer circulation in the bloodstream. The hydrodynamic diameter of particles (~150 nm) prevents the particles from passing through the tight junctions within normal blood vessels or being excreted by the kidney [24]. As a result, AuroShell Particles passively and selectively accumulate and are retained within the tumor perivasculature for at least 48 h in an animal model [25] due to a tumor’s dense and leaky microvasculature and defective lymphatic clearance, a process known as Enhanced Permeability and Retention (EPR) [24,26].

Moreover, AuroShell Particles have optical properties that can be tuned in the NIR window where water in tissue and hemoglobin have low absorption [27] by adjusting their core-to-shell ratio [28]. NIR photons propagate into vascular tissue with a limited penetration depth of 3–5 mm [29]. Under NIR laser illumination, AuroShell Particles scatter and absorb NIR light. Incident photons excite AuroShell Particles, which transduce the absorbed optical energy into thermal energy, producing ablative heat that conducts into the surrounding tissues, while the scattered photons tend to even out inhomogeneities in light distribution within tissue and are available for subsequent absorption [30]. Treatment dosimetry was established with three minutes of continuous NIR illumination (810 nm) at 5.7 W emitted from a 1.8 cm long isotropic optical fiber diffuser (OFD) as sub-ablative to nanoshell-free tissues and localized to a millimeter scale.

The focality of AuroLase Therapy is achieved by the tumor-specific accumulation of nanoshells and by combining multiparametric magnetic resonance imaging (mpMRI) with MR/ultrasound fusion targeting for high tumor detection sensitivity and precise targeting [15]. Transrectal ultrasound (TRUS) imaging alone cannot render intra-prostatic anatomical details and does not easily differentiate tumors from prostate tissues within the gland, leading to a high risk of missing cancer, compromising the disease diagnosis and treatment. A combination of MRI with TRUS for guided therapy targets lesions with high sensitivity and specificity using real-time ultrasound imaging [31]. Furthermore, mpMRI provides anatomic and functional images to identify the tumor’s exact location and conformation by generating a 3-dimensional (3D) model relative to the entire prostate gland. This 3D model is “fused” with real-time ultrasound imaging, permitting the model to be manipulated and conform to the real-time ultrasound image. Thus, the modeled tumor size and location can be “visualized” in the ultrasound field where it may not otherwise be visible, permitting very accurate biopsy sampling [32] and optical fiber placement in a conventional operating room suite. The combination of mpMRI with MR/US fusion targeted biopsy platforms has dramatically enhanced the ability to identify and target suspicious lesions, resulting in better differentiation between malignant and non-malignant tissues and tumor staging and targeting [31].

Newly-published clinical data have demonstrated the effectiveness of AuroLase Therapy to precisely ablate low-to-intermediate-risk localized PCa with 94% success in 16 patients, with no significant difference in International Prostate Symptom Score (IPSS), Sexual Health Inventory for Men (SHIM) or urinary Quality of Life (QoL) [15]. To date, 102 PCa patients have been treated in Phase I/II trials of AuroLase Therapy, with all one-year follow-up evaluations completed by the end of 2022. Here, we present a single clinical case as illustrative for the general and intended course of the treatment timeline as it developed from small and large animal experiments [33,34] through the clinical trials [15,16].

Most patients entering this study began with a detectable increase in their PSA scores. Multiparametric-MRI-directed biopsies assessed the cancer diagnosis, treatment planning, and therapy assessment. Dynamic Contrast Enhanced (DCE) MRI with measurements of contrast agent (CA) wash-in/out color mapping and its most quantitative pharmacokinetic parameter, K^trans^ (CA volume transfer constant), were highlighted for their potential to effectively demarcate areas with absent perfusion due to the controlled ablation treatment. A combined analysis of the K^trans^ and apparent diffusion coefficient (ADC) map, a quantitative parameter for water molecule diffusion within tissues, was implemented for treatment response assessment during the first year of follow-up post-AuroLase Therapy.

## 2. Detailed Case Description

**Figure 1:**
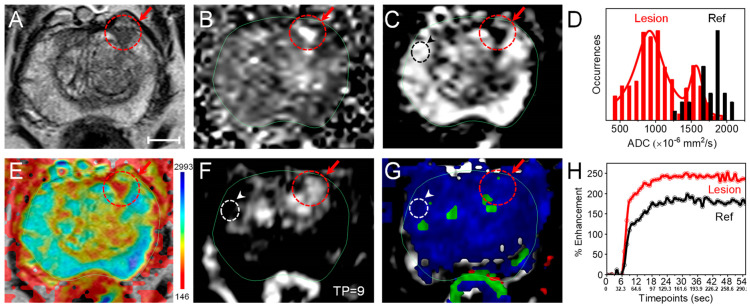
**Multiparametric MRI data of a 69-year-old male with focal prostate cancer on the left Anterior Peripheral Zone (PZa) of the prostate midgland; PSA = 4.57 ng/mL, prostate size: (4.6 cm × 3.6 cm) × 4.1 cm (34.4 cc).** (**A**–**C**) The lesion site appeared hypointense with an erased charcoal appearance on an axial *T*_2_w MRI (**A**), hyperintense on an axial high b-value diffusion-weighted image (DWI) (**B**), and focally hypointense on an axial apparent diffusion coefficient (ADC) map (**C**). (**D**) ADC value distributions at the lesion site (Lesion, red) in comparison to healthy PZa tissues (Ref, black) highlighted on the ADC map (**C**) with dashed red and black circles, giving an ADC value (median ± SD, range) of 1007 ± 348 (446–1975) × 10^−6^ mm^2^/s and 1794 ± 186 (1200–2028) × 10^−6^ mm^2^/s processed by Invivo DynaCAD, respectively. (**E**) An axial *T*_2_w image with an ADC map overlay (ADC values range: 146–2993 × 10^−6^ mm^2^/s) highlights the restricted diffusion area corresponding to the region of interest on the *T*_2_w MRI. (**F**) An axial background-subtracted *T*_1_w MRI taken at a timepoint = 9 of the dynamic contrast-enhanced (DCE) MRI sequence with a gray color revealed the early contrast agent (CA) enhancement at the lesion site (dashed red circle and arrow) compared to healthy tissues (Ref, dashed white circle and an arrowhead) in the right PZa. (**G**) An axial background-subtracted *T*_1_w MRI (timepoint = 69) with a DCE color map overlay (K^trans^/V_e_). (**H**) Percentage of contrast enhancement kinetics curve of the DCE MRI at the lesion (red) and Ref (black) at different timepoints. The lesion was assigned as PI-RADS 4 (Gleason 6; Grade Group 1). Note: the slices of the *T*_1_w MRI and functional MR images (DWI, ADC, and DCE) correspond to the same slice shown in the *T*_2_w MRI (slice thickness: 3 mm; external magnetic field: 3 T, scale bar = 10 mm).

**Diagnosis and staging.** A 69-year-old man with a PSA level (density) of 4.57 ng/mL (0.13 ng/mL/cc) was diagnosed with PCa. An anatomical MR image (axial *T*_2_w MRI, Figure 1A) revealed a suspicious lesion with an “erased charcoal” appearance, homogenously hypointense, and partially circumscribed margins on the left Anterior Peripheral Zone (PZa) of the prostate mid-gland. Functional MRI images such as the Diffusion Weighted Image (DWI) and ADC map were used to look at the water molecule restrictive diffusion due to high tumor cellularity level or/and inflammatory response. The high cellularity level at the lesion site appeared focally hyperintense on a high magnetic field gradient (b-value) DWI while focally markedly hypointense on the ADC map with lower ADC values in comparison to healthy PZ tissue (Figure 1B–D). An overlay of the ADC map over the *T*_2_w MRI (Figure 1E) highlights the lesion in the PZa, giving a volume of 0.43 cc [0.7 cm × 0.6 cm (in-plane), ×1.2 cm (extent)], which involved ~1.2% of the prostate gland volume (34.4 cc). On the DCE MRI (gray and color maps) and enhancement kinetic plot (Figure 1F–H), the lesion had an early and higher contrast enhancement versus normal PZ tissues. Based on Prostate Imaging–Reporting and Data System (PI-RADS) version 2.1 [35], the lesion was scored as PI-RADS 4 (highly likely clinically significant cancer with a <1.5 cm greatest dimension). Biopsies confirmed prostatic adenocarcinoma with a Gleason Score 6 = 3 + 3 (International Society of Urological Pathology Gleason Grade Group (ISUP GGG 1)). Furthermore, inconsistent areas of restricted diffusion throughout the PZ were detected and corresponded with striated *T*_2_ signals consistent with prostatitis. The central gland consisted of glandular and stromal hyperplastic nodules, but was free of lesions.

**Figure 2:**
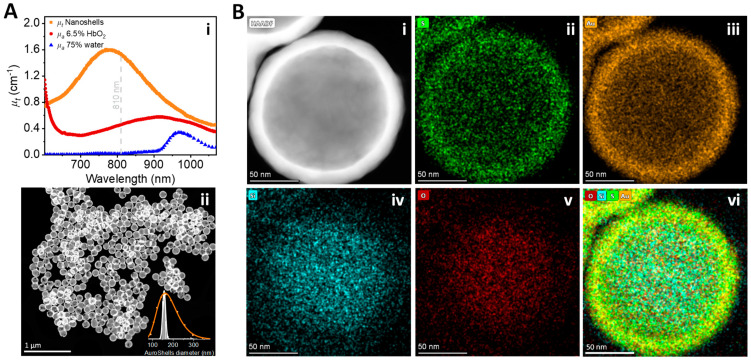
Characterization of an AuroShell Particle. (**Ai**) An absorption spectrum of an aqueous suspension of AuroShell^®^ Particles in the near-infrared range with a maximum absorption peak near ~780–800 nm at concentrations expected to be at the targeted tumor (orange), expected hemoglobin concentration of 6.5% by volume (red), and expected water concentration of 75% by volume (blue). (**Aii**) A high-resolution Scanning Transmission Electron Microscopy (HR-STEM) of AuroShell Particles with a high-angle annular dark-field imaging mode. The insert represents the particle diameter distributions with Gaussian fit functions (white line), giving a mean±SD of 156 ± 7 nm (472 analyzed particles). The DLS measurement of the bulk sample of the AuroShell Particle suspension (orange circles) gives an average particle hydrodynamic diameter of 156 nm. (**B**) A HR-STEM image of a single AuroShell Particle with the STEM-HAADF imaging mode (**i**) and its elemental mapping images correspond to (**ii**) Sulfur (S), representing the thiol functional group on the PEG chain which has a covalent bond with the gold shell, (**iii**) Au- representing the gold shell, (**iv**,**v**) Si- and O-, representing the silica dioxide core, and (**vi**) a composite image of all of the elements.

**Figure 3:**
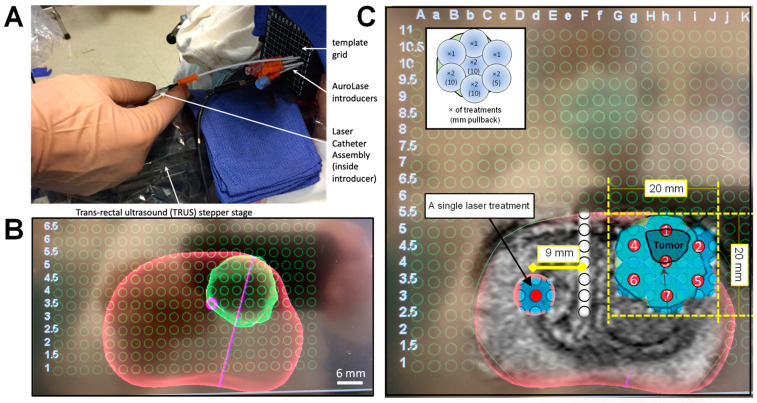
**Laser treatment sequence under live MR/ultrasound fusion (MR/US) guidance.** (**A**) The AuroLase^®^ procedure showing a Laser Catheter Assembly inside the cannula of the trocar introducer, in which the lateral position is fixed by the transperineal template grid. (**B**) A cropped screenshot from the UroNav screen showing the 3-dimensional (3D) prostate surface rendered image post-MR/US after the TRUS and template grid had been registered in 3D. The screenshot highlights the focal ablation volume (green: lesion plus ~7 mm margin) within the prostate gland (red). (**C**) An axial *T*_2_w MR image overlaid on the corresponding ultrasound image, visualizing how the two imaging modalities assist in localizing the tumor position in 3D space relative to the prostate gland. The prostate (red), ablation zone (tumor + margin: dark and light blue), and laser catheter insertion positions (numbered circles) are superimposed on the grid and represent the laser catheter insertion sequence. At the end of the treatment, an additional treatment [control burn] was performed in the contralateral hemisphere ~0.9 cm far from the urethra (U). The inset shows the number of laser treatments at each position in the treatment zone and the pullback distance (in mm) as needed to cover the longitudinal space of the targeted ablation volume. Based on this plan, the expected ablation dimensions were 2.0 cm × 2.0 cm (in-plane), 2.8 cm (extent).

**AuroLase**^®^ **Therapy.** The tumor was focal and contained within the confines of the prostate capsule, fulfilling the AuroLase Therapy trial inclusion requirements [15] (method section). The patient underwent the two-day AuroLase Therapy, infused with AuroShell^®^ Particles (Figure 2) on one day and laser treated the following day (Figure 3).

Seven 14-gauge catheter trocars with 6-8 mm lateral spacing were inserted through a template grid aligned with the transverse plane of the prostate and used to provide insertion tracts through the perineum for 11 laser treatments with a transperineal approach. The treatment covered the ~0.43 cc tumor volume plus a ~7 mm margin. Four of the laser treatments were conducted after pulling back the optical fiber ~5–10 mm toward the apex part of the prostate, covering the longitudinal aspect of the tumor. Figure 3 is the UroNav screenshots during the live MR/US transperineal laser treatment, demonstrating the laser placement sequence and the ablation zone it is expected to form.

The Method section (Section 5) and Supplementary Materials, Figures S1–S3 cover laser delivery system, ablation zone generated from the 1.8 cm optical fiber diffuser, and treatment planning (introducer insertion sequence).

**Figure 4:**
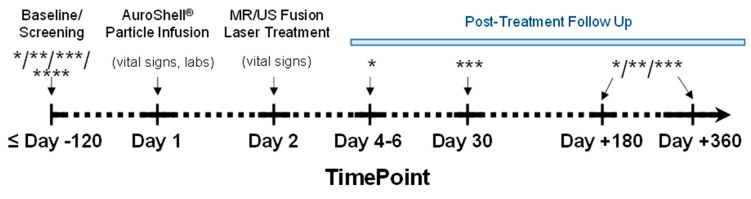
**Clinical AuroLase**^®^ **Therapy timeline.** * mpMRI; ** MR/US fusion transperineal biopsy; *** IPSS, SHIM, EPIC-26, vital signs, physical examination, labs; **** others (demographics, medical history, etc.).

**AuroLase**^®^ **Therapy Assessment.** The patient was treated and released on the same day with no serious adverse events after successful voiding and was followed up (mpMRI scans, PSA, MR/US transperineal biopsies, etc.) at <10 days, 6 months, and 1 year post-treatment (Figure 4). The primary mpMRI sequences used for therapy assessment were as follows: (1) anatomical images (*T*_2_w MRI), and (2) functional images (DWI and ADC, DCE wash-in/out color, and K^trans^ maps) for any sign of treatment-related changes or tumor recurrence.

***Follow up on Day 5.*** Multiparametric MRI assessment of the thermal damage was conducted 48–96 h post-AuroLase to map the thermal ablation zone generated as a result of the treatment and compare it to the original treatment plan (Figure 3).

**Figure 5:**
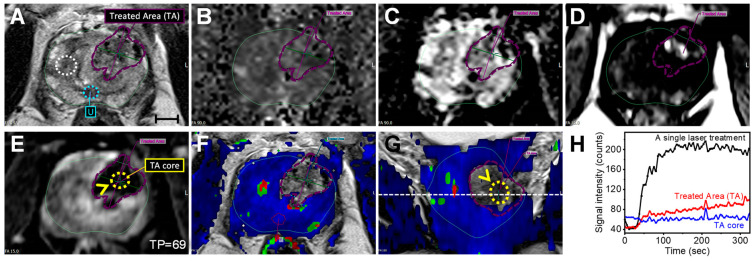
**AuroLase**^®^ **Therapy assessment with mpMRI on Day 5.** (**A**–**E**) The treated tumor area (dashed purple line) had heterogeneous and low signal intensities on an axial *T*_2_w MRI (**A**), low signal intensities on an axial high b-value DWI (**B**), low ADC signals on an axial ADC map based on DWI with b-values of 50 and 800 s/mm^2^ (**C**), high intensity on an axial *T*_1_w MRI without background subtraction and before *T*_1_ contrast agent injection (**D**), and absent signal enhancement on an axial background-subtracted *T*_1_w MRI, the last *T*_1_w MRI acquired (TP = 69) during the DCE sequence acquisition (**E**). (**F**) An axial *T*_2_w MRI with a DCE (contrast agent wash-in/out) color map overlay corresponds to the dashed white line across the coronal plane in (**G**). The two planes highlight the treated area with a void space within the prostate gland capsule. (**H**) A CA signal enhancement kinetic at three regions of interest: (1) the treated tumor (red line), (2) its core (blue line), and (3) a single laser treatment at the right side of the prostate gland (black line) corresponds to the dashed white circle in (**A**). Additional color coding: solid green line—the prostate capsule, yellow arrowhead/dashed circle—the treated tumor core, and cyan dashed circle—the urethra (U). Note: the treated area was manually created on the DCE color map and was propagated to the rest of the images for further analysis (scale bar = 10 mm).

**Figure 6:**
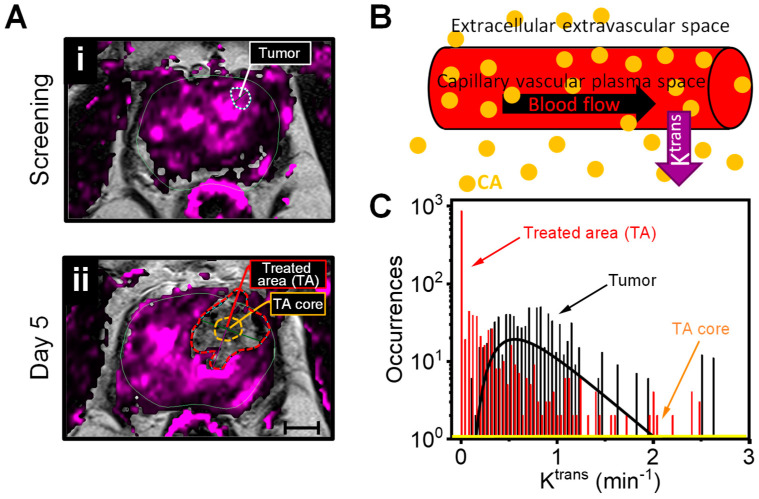
**K^trans^ for a quantitative evaluation of AuroLase**^®^ **Therapy effectiveness to ablate prostate tissue.** (**A**) Axial *T*_2_w MR images with a K^trans^ color map overly at baseline (**i**) and Day 5 post-treatment (**ii**), highlighting the lesion (left PZa midgland, dashed white line), treated area (TA, dashed red line), and the TA core (dashed orange line). (**B**) Schematic representation of the volume transfer constant, K^trans^, representing the diffusion of the intravascular *T*_1_ MRI contrast agent (CA) into the extracellular extravascular space. (**C**) K^trans^ distributions on a logarithmic scale at the treated area (red) with zero occurrences at its core (orange line) in comparison to the lesion (black). The solid black line is a LogNormal fit of the tumor K^trans^ distribution, giving a median ± Log-SD of 0.75 ± 0.5 min^−1^.

As a consequence of the treatment, the prostate capsule, denoted with a solid green line (Figure 5), was found to be distorted at the treated side of the prostate due to expected post-treatment edema, increasing the prostate volume by ~4.4% (from 34.4 cc to 35.9 cc). The tumor-treated area presented with a dashed purple line in Figure 5A–D appeared heterogeneous with low signal intensities on the *T*_2_w MRI, DWI, and ADC map and with high signal intensities on the *T*_1_w MRI (without a *T*_1_ CA and background subtraction) due to necrotic tissue and the presence of a hemorrhage, consistent with what is expected post-treatment [36,37,38]. Furthermore, a cavity with no signal enhancement was evident within the treated area on the DCE MRI (axial background-subtracted *T*_1_w MRI at TP = 69: Figure 5E and axial/coronal *T*_2_w images with DCE color map: Figure 5F,G). The ablated area outlined with a dashed purple line gave an ablated volume of 5.01 cc [2.6 cm × 2.0 cm (in-plane), 2.7 cm (extent)]. The ablated area was contained entirely within the confines of the prostate capsule and was convincingly conformal to the planned ablation zone dimensions [2.0 cm × 2.0 cm (in-plane), 2.8 cm (extent)] (Figure 3). A curve-type analysis of DCE MRI, Figure 5H, shows the signal enhancement at a region of interest before, during, and after the injection of a *T*_1_ CA into the patient. A weak signal enhancement at the treated area (red line) was detected, with no signal enhancement at its core (blue line). The volume transfer constant map, K^trans^, which is the most quantitative parameter for DCE MRI representing the diffusion of the intravascular MRI CA into the extracellular extravascular space, reveals a void of space in the treated area with high occurrences (logarithmic scale) at lower K^trans^ values than that for the tumor (0.75 ± 0.5 min^−1^; median ± Log-SD) (Figure 6). No occurrences were detected at any K^trans^ value at the center of the treated area (Figure 6C, orange line), consistent with what is expected at locations with absent perfusion due to a controlled ablation site.

A single additional treatment was conducted under identical illumination conditions (810 nm, 1.8 cm OFD at 5.7 W for 3 min) at the contralateral side of the prostate close to the PZ, where no tumor was MRI-visible at screening. On Day 5 mpMRI, it was challenging to precisely determine the location of this treatment because signal enhancement was evident on the DCE color map across the right hemisphere of the prostate, where this treatment occurred. However, the location of this treatment site relative to the treated lesion and the urethra was predictable based on the treatment plane (Figure 3 and Supplementary Materials, Figure S4). This treatment area had low signal intensity on the axial *T*_2_w MRI (Figure 5A, dashed white circle), corresponding to a normal contrast enhancement signal (Figure 5H, black line) similar to the trend detected in healthy PZ tissues at screening (Supplementary Materials, Figure S5). This treatment validates that the chosen laser dosimetry is below the level that would cause MRI-visible coagulative necrosis.

**Figure 7:**
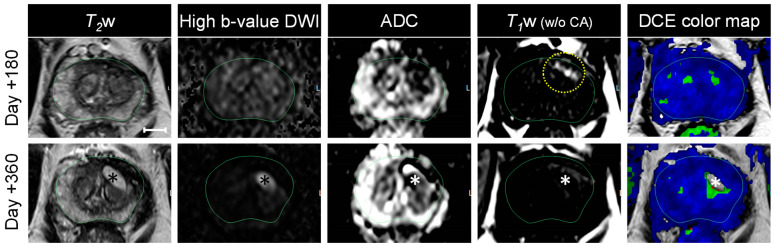
**Six- and twelve-month follow-up mpMRI post-AuroLase**^®^ **Therapy.** Axial mpMRI on Day +180 and Day +360, covering the *T*_2_w MRI, high b-value DWI, ADC map, *T*_1_w MRI (without the *T*_1_ contrast agent (CA) and background subtraction), and *T*_2_w MRI with the DCE color map overlay (from left to right, scale bar = 10 mm). The MRI slices at the two follow-up timepoints correspond with the slices presented at screening (Figure 1) and Day 5 (Figure 5) performed under a 3T external magnetic field scanner. Four core biopsies were taken from the treated lesion (left PZa) at six months and confirmed benign–negative for malignancy. Evidence of hemorrhage due to treatment was still evident on Day +180 mpMRI (dashed yellow circle) and entirely resolved at one year. At one year post-treatment, 12 standard-core biopsies were taken (6/6 of the biopsies taken from the left hemisphere of the prostate, including the treated lesion) were confirmed negative for malignancy with evidence of focal acute and chronic inflammation. Treatment-related cystic changes (*) appeared bright on *T*_2_w MRI and DWI (high b-value), dark on the ADC map and *T*_1_w MRI (*w*/*o* the CA), and a lack of enhancement on the DCE color map. From the right hemisphere, where no MRI-visible lesions were detected on screening, six biopsies were taken, in which one biopsy confirmed atypical small acinar proliferation (ASAP) at the right PZa, one biopsy confirmed PCa (ISUP GGG 1) at the right transition zone, and the remaining four biopsies were benign–negative for malignancy.

***Follow up on Day +180 and Day +360.*** Additional follow-up timepoints were conducted at six months (Day +180) and one year (Day +360) post-treatment by performing mpMRI and targeted MR/US fusion transperineal biopsies. Figure 7 summarizes the primary MRI sequences relevant for treatment evaluation. A direct comparison of the mpMRI data at these timepoints with those at screening and Day 5 post-therapy is shown in Supplementary Materials, Figure S6.

The treated area still had heterogeneous and low signal intensities on the *T*_2_w MRI and ADC map on Day +180 mpMRI (Figure 7), which developed to a dark band-like/scar appearance by Day +360, suggesting fibrosis. A residual bright signal was still evident on the *T*_1_w MRI (without contrast and background subtraction) on Day +180, likely a residual hemorrhage due to the treatment, which was entirely resolved by Day +360. Furthermore, there were no visible areas with absent perfusion on the DCE color map on Day +180. On Day +360, a fluid-filled cavity was evident (Figure 7, *) with noticeable hyperintense signals on the *T*_2_w MRI and DWI, and a hypointense signal on the *T*_1_w (without the CA and background subtraction), and a lack of perfusion on the DCE color map.

**Figure 8:**
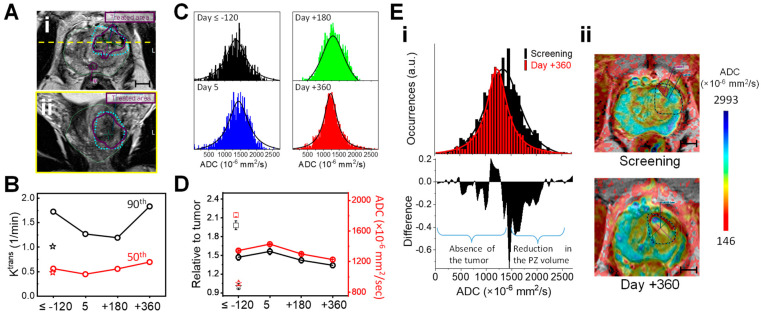
**K^trans^ and ADC values for response assessment to AuroLase**^®^ **Therapy.** (**A**) An axial *T*_2_w MRI (**i**) and its corresponding image in the coronal plane (**ii**) along the dashed yellow line on Day 5 highlighting the region of interest (ROI, a dashed cyan line) covering the left anterior quartersphere and extending from the base to the apex of the prostate gland while excluding the urethral track (U). (**B**) The 90th (black) and 50th (red) percentile K^trans^ values of the ROI at screening (Day ≤ −120) and post-therapy (Day 5, +180, and +360). (**C**) The ROI’s ADC distributions at the same timepoints. (**D**) The ROI’s ADC median values ± SEM (range) × 10^−6^ mm^2^/s of the ADC distributions fitted with Voigt functions processed with OriginPro 2022, giving ADC values of 1345 ± 9 (23–2526) at screening, 1429 ± 9 (32–2535) on Day 5, 1300 ± 7 (298–3576) on Day +180, and 1227 ± 3 (33–2728) on Day +360 (red circle symbol). The data in black are normalized by the tumor (star symbol). The treated lesion (star symbol) had a K^trans^ value of 1.24 min^−1^ and 0.72 min^−1^ at the 90th and 50th percentiles, respectively, and an ADC median value ± SD (range) × 10^−6^ mm^2^/s of 1007 ± 348 (446–1975) in comparison to a healthy PZ tissue (square symbol) at the right PZa with an ADC value of 1794 ± 186 (1200–2028) which was obtained from Figure 1D. (**Ei**,**Eii**) A comparison of the ADC distributions of the ROI at screening (black) and one year post-treatment (red) and their difference (**i**) and their corresponding axial *T*_2_w MR images with an ADC map overlay (**ii**, scale bar = 1 cm).

**K^trans^ and ADC values for quantitative analyses.** Post-AuroLase^®^ Therapy, the untreated tissues are expected to reposition and adjust to the treatment-related changes at the ablated zone. For this case, however, it is not likely for the tissue located at one quarter of the prostate gland to move to a different quadrant. Thus, the post-treatment changes at the ablated zone can be tracked by creating a large region of interest (ROI, dashed cyan line in Figure 8A), covering most of the left anterior quartersphere of the prostate gland from the base to the apex while excluding the urethra and the neurovascular bundle to avoid errors in the K^trans^ and ADC calculations processed with Invivo DynaCAD. Figure 8B–D summarize the K^trans^ and ADC trend values during the first year of follow-up. On Day 5 post-therapy, a decrease was observed in the 90th percentile K^trans^ value from 1.723 cm^−1^ at the screening to 1.267 cm^−1^ while an increase was observed in the median ADC values ± SEM (range) from 1345 ± 9 (23–2526) × 10^−6^ mm^2^/s at screening to 1429 ± 9 (32–2535) × 10^−6^ mm^2^/s. At six months, the 90th percentile K^trans^ value was still low (1.192 cm^−1^), and the ADC value decreased further to 1300 ± 7 (298–3576) × 10^−6^ mm^2^/s with increased occurrences at higher ADC values, giving a broader ADC distribution. At one year post-ablation, the 90th percentile K^trans^ value recovered close to its pretreatment value. Furthemore, a decrease in the ADC distribution line width (Figure 8C) gave an ADC median value of 1227 ± 3 (33–2728) × 10^−6^ mm^2^/s. Figure 8E compares the changes in the ADC distribution and the ADC map at one year post-treatment versus at screening, providing a visual illustration of the treated PZ degradation with (1) the absence of high ADC values, which are unique for healthy PZ tissues (Figure 1D—black distribution), giving a negative difference as a result of subtracting the ADC distribution at screening from that at one year post-ablation (Figure 8Ei) and (2) the absence of cyan color, representing high ADC values, on the left anterior quartersphere of the prostate gland at one year post-treatment in comparison to the screening *T*_2_w/ADC infusion MRI (Figure 8Eii). These findings suggest that most of the treated PZ, where the tumor was located, was degraded by one year post-AuroLase Therapy.

**Prostate volume, PSA, biopsies, and other types of assessments.** At one month, six months, and one year post-treatment, the prostate volume, PSA level, MR/ultrasound fusion transperineal biopsies, and other documents were filed for every patient in the clinical Phase I/II device trial to verify the treatment resolution. Baseline and follow-up PSA scores, biopsy results, prostate volumes, and self-assessments (IPSS, SHIM) for the patient are summarized in Table 1. Post-AuroLase^®^ Therapy, the prostate volume had decreased by 28% and 24% from 34.4 cc at baseline to 24.8 cc at six months and 26.1 cc at one year, respectively, as processed with Invivo DynaCAD. At one year, the PSA (density) decreased by 53% (38%) from 4.57 ng/mL (0.13 ng/mL/cc) at baseline to 2.16 ng/mL (0.08 ng/mL/cc). Multiple MR/US fusion transperineal biopsies were collected from the treated site, confirming benign–negative for malignancy at six months and one year. Twelve standard core biopsies at one year confirmed focal acute and chronic inflammation at the treated area, consistent with cystic necrosis (Figure 7, *). For the IPSS, which assesses urinary function/symptoms as interpreted by the patient, the patient scored 10, 5, 5, and 9 at baseline, +20 days, +182 days, and +373 days, respectively. Additionally, for the SHIM, which measures the subjective performance of erectile function, the patient scored 19, 20, 20, and 14 at baseline, +20 days, +182 days, and +373 days, respectively.

IPSS (International Prostate Symptom Score): assesses urinary function/symptoms as interpreted by the patient (0–7: mild, 8–19: moderate, and 20–35: severe symptoms); SHIM (Sexual Health Inventory for Men): measures the subjective performance of erectile function (1–7: severe erectile dysfunction (ED), 8–11: moderate ED, 12–16: mild to moderate ED, 17–21: mild ED, and 22–25: no signs of ED)

## 3. Discussion

Various focal therapies have been evolving to treat localized PCa utilizing high laser powers, high-intensity focused ultrasound, high-pressure steam, or liquid gas [8]. Unlike AuroLase^®^ Therapy, unnecessary damage to healthy tissue may be caused as a result of the inaccurate positioning of tools due to detection limitations and the ablation zones’ shape defined by the emitting device. AuroLase Therapy leverages the potential of non-toxic nanoparticles, which passively accumulate and are retained in the tumor microenvironment [16,34], to induce homogeneous ablative heat under nominally sub-ablative laser powers. As a result, localized tissue damage selectively occurs to the tumor while sparing healthy (nanoshell-free) tissue. Thus, inaccurate positioning of the laser fiber should not cause significant harm to tissues lacking nanoshells.

A 69-year-old male was diagnosed with localized low-risk PCa at the left PZa mid-gland and treated with AuroLase Therapy under live MR/ultrasound fusion imaging guidance. The therapy combines mpMRI (anatomical and functional MRI) with a transperineal approach method for better diagnosis, targeting, and treatment evaluation. DWI and ADC mapping evaluate the random diffusion of water molecules in various tissues due to high cellularity levels in tumors, for instance, providing higher signal intensities on the high b-value DW image and lower signal intensities on the ADC map relative to healthy tissues (Figure 1B,C). Unlike DWI, the ADC map reflects only the diffusion of water molecules and is independent of artifacts in the baseline *T*_2_w MRI of the DW images. Furthermore, the ADC values for malignant PZ were found to be statistically significantly different from the ADC values for non-malignant PZ (*p* = 0.0001) [39] and inversely correlated with the tumor Gleason score (*p* < 0.001) [40] with ADC values < 900 × 10^−6^ mm^2^/sec to be considered clinically significant cancer [41]. This aligns with the treated lesion (PI-RADS 4) having a median ADC value ± SD of (1007 ± 348; min = 446) × 10^−6^ mm^2^/s, which is lower than a healthy PZ (1794 ± 186; min = 1200) × 10^−6^ mm^2^/s processed with Invivo DynaCAD. Utilizing OriginPro (2022), the lesion distribution (solid red line in Figure 1D) was deconvoluted into two Voigt functions with ADC values (mean ± SEM) of 915 ± 30 × 10^−6^ mm^2^/s and 1571 ± 34 × 10^−6^ mm^2^/s, providing a lower ADC for the tumor within the delineated area. The ADC values and the earlier and higher contrast enhancement (Figure 1D,F,H) assist in determining the lesion volume and grade, which was confirmed as prostatic adenocarcinoma with an ISUP GGG 1.

For the AuroLase Therapy, the patient was infused with AuroShell^®^ Particles the day before the laser treatment. The ~150 nm hydrodynamic diameter nanoshells prevent excretion by the kidney [24,42], and their surface functionalization with hydrophilic PEG reduces phagocytosis and bypasses the reticuloendothelial system [43]. Consequently, AuroShell Particles circulate in the bloodstream with a 3–6 h half-life [25,34], increasing the accumulation and retention within the tumor through the EPR effect. About 0.15% (range: 0.028% to 0.816%) of the injected AuroShell Particles (corresponding to 8.28 µg Au/g tissue and 4.24 × 10^8^ nanoshells/mL) are expected to accumulate at the prostate tumor site [15], which is within the expected percent range of nanoparticles delivered to tumors found in the literature [44]. The accumulated nanoshells induced efficient thermal damage under NIR illumination (3 min at 5.7 W with an 810 nm continuous laser coupled from a 1.8 cm long ODF).

The key to a successful treatment is effective treatment planning. This was accomplished by fusing MRI data with live ultrasound imaging, allowing targeting and tracking of the ablation process. For accurate tumor targeting, the anterior fibro-muscular stroma part was included in the prostate segmentation on the MRI so that the generated 3D rendered prostate volume could be aligned with that generated on the live ultrasound image, providing anatomical details. Furthermore, a ~7 mm margin was added to the targeted ablation zone in addition to the tumor volume visualized with mpMRI (Figure 3), taking into account that *T*_2_w MRI underestimates the size of the tumor, especially along the base–apex axis [45] and for small radiologic tumor size and low PI-RADS v2 scores [46].

The first follow-up mpMRI measurement was conducted on Day 5 (48–96 h post-treatment), allowing sufficient time for complete coagulative necrosis before tissue reconfiguration by lytic action. This stage assists in validating the therapy plan, tumor targeting, and laser dosimetry. Even though there is no standard timing on when to conduct early post-focal treatment monitoring in regular therapeutic practice, it is recommended to be performed from 48 h post-treatment to a week to assess the treatment and determine whether the tumor volume was undertreated. Scanning at a longer time (>10 days post-treatment) will complicate the treatment evaluation due to the penetration of the surrounding granulation tissues into the necrotic tissues, forming a double rim of enhancement on the DCE with a smaller treated volume in addition to the presence of various inflammatory changes. Localized edema, hemorrhage, glandular loss, and fibrosis were evident on the mpMRI at this timepoint, with the bright area on the *T*_1_w MRI (without background subtraction and *T*_1_ CA) that correlate with the heterogeneous decrease in the *T*_2_w MRI signal intensities at the treated site (Figure 5A,D). A sign of hemorrhage was still evident in the 6 month mpMRI data (Figure 7), by the residual of the bright spot on the *T*_1_w image (without background subtraction and *T*_1_ CA), and was undetectable by one year. Even with much less invasive procedures such as prostate biopsy collection, hemorrhage is detectable by MRI for an extended period, up to 60 days post-biopsy in some cases [47].

Even though some changes were visible on the *T*_2_w MRI, including the heterogeneous decrease in its signal intensities, which mimics the dark signal in a tumor, it is insufficient to visualize the ablation zone and determine its quality. Functional MRI modalities (DWI, ADC mapping, and DCE MRI) are valuable and can potentially assess the treatment efficacy and detect cancer recurrence with high sensitivity and specificity [48,49,50,51,52]. During the first seconds of laser illumination, enhanced perfusion is expected at the treated site due to the increased permeability of the tumor vessels (tumor macrovascular pore size) [53]. After laser treatment, a reduction and even a complete shutdown of the tumor vasculature is expected, resulting in no contrast agent uptake [15]. This was evident with the appearance of the void space on the DCE color map post-treatment and consistent with weak/no signal enhancement at the treated area/core (Figure 5F–H). Such results were expected in an area with absent perfusion due to a controlled ablation site [15,54,55]. Bomers et al. have correlated volumes that have nonenhanced signal intensity on DCE (V_DCE_) post-MRI-guided focal laser ablation with the necrotic volume (V_n_) observed on the whole-mount histopathology section post-radical prostatectomy [56]. A significant correlation was reported with a median volume ratio (V_n_/V_DCE_) of 0.80 (range: 0.4–2.09) and a Pearson Correlation Coefficient of *r* = 0.94 (*p* = 0.018) [56]. Thus, most of the measured nonenhanced signal on the DCE color map (Figure 5F,G), ~14% (5.0 cc) of the prostate volume could be considered necrotic tissue contained within the prostate capsule with no damage to the surrounding soft tissues and muscles with dimensions as planned. The single laser treatment performed on the prostate gland’s right hemisphere was insufficient in producing MRI-detectable thermal damage; instead, a sign of scarring was observed on the *T*_2_w MRI with a normal contrast enhancement (Figure 5A,H), suggesting that the laser dosimetry used is sub-ablative, insufficient to cause MRI-detectable tissue damage.

Monitoring the changes in the K^trans^ (vascular permeability and perfusion) and ADC (water diffusion restriction) values may assist in evaluating treatment efficacy and providing quantitative analyses [52,57]. The absent perfusion observed on the Day 5 DCE MRI could be correlated with the void that appears on the K^trans^ map (Figure 6A) and consistent with the low K^trans^ values with zero occurrences of any K^trans^ values at the core of the treated area (Figure 6C). Pre-clinical data have shown that shutting down tumor vasculature via acute inhibition of vascular endothelial growth factor A signal transduction in mice bearing PC-3 human prostate adenocarcinoma xenografts leads to a reduction in the tumor K^trans^ values [58]. In another study, the K^trans^ value decreased from 0.44 ± 0.25 min^−1^ to 0.29 ± 0.17 min^−1^ while the ADC value increased from 1090 ± 230 × 10^−6^ mm^2^/s to 1240 ± 280 × 10^−6^ mm^2^/s (mean ± SD) three months after the MRI-guided focal laser ablation of the PCa [52]. Furthermore, the hemorrhage on Day 5 could be correlated with the increase in the median ADC values (Figure 8D). Higher ADC values were significantly associated with higher hemorrhagic scores post-biopsy [47].

At six months post-therapy, the treated area may have partially degraded. This is suggested by the contrast enhancement recovery detected on the DCE color map (Figure 7) and its K^trans^ map (Supplementary Materials, Figure S6). Moreover, an increase in occurrences at higher ADC values was detected at this time point (Figure 8C). Such findings are consistent with Song I et al., in which a statistically significant increase was observed in the ADC mean value ± SD (range) of the overall PCa-treated area from 1000 ± 190 (730–1580) × 10^−6^ mm^2^/s to 1610 ± 270 (890–2460) × 10^−6^ mm^2^/s one to five months post-radiotherapy of PCa at 3 Tesla (*p* < 0.001) [59]. Such an increase in the occurrences of high ADC values post-treatment could be treatment-related changes [52,59], including coagulative necrosis, suggesting that the treated area was not entirely degraded even though a normal contrast enhancement was observed by 6 months.

At 1 year post-therapy, the necrotic tissues have entirely degraded. The recovery of the K^trans^ values (Figure 8B) could be indirectly related to necrotic tissue degradation because necrotic tissues have lower K^trans^ values than healthy tissue. The likelihood of tissue degradation was also supported by the absence of high ADC values that appeared at six months (Figure 8C) and by the absence of the PZ at the treated area in Figure 8Eii at one year that originally has high ADC values (Figure 1D—black distribution). The ADC median value decreased to a value within the expected ADC values reported for non-malignant tissue in the transition zone (1212 ± 0.0002 × 10^−6^ mm^2^/s, *p* < 0.0001) [60]. Moreover, the absence of low ADC values, which are contributed to by the tumor (Figure 1D—red distribution), presented with a negative difference in Figure 8Ei, indicating that the treatment was successful and confirmed with negative targeted MR/US fusion transperineal biopsies at 6 months and 1 year post-AuroLase therapy. Of note, this patient developed a cyst, a fluid-filled cavity, evident on the one-year mpMRI (Figure 7, *), apparently due to an infection that arose subsequent to a biopsy and was unrelated to the treatment.

In addition to the non-perfused volume coverage and negative biopsies within and around the treated tumor, other effective endpoints were considered for this therapy, including PSA reduction and stability and prostate volume reduction. The patient’s PSA and prostate volume were reduced post-treatment relative to the baseline, in agreement with the reduction reported for the first 16 patients treated with AuroLase^®^ Therapy [15]. Post-AuroLase Therapy, the 1-year PSA value is expected to be detectable because a large portion of the prostate gland will be untreated with this therapy, secreting PSA.

## 4. Conclusions

Focal therapies, including cryoablation, HIFU, TULSA, brachytherapy, IRE, LITT, VTP, and AuroLase Therapy, can potentially treat localized PCa, preserving baseline functionality. As distinct from other therapies, AuroLase^®^ Therapy—a nanoparticle-assisted therapy─ harnesses the potential of nanoparticles to passively accumulate and retain in the PCa micro-environment. The therapy causes selective ultrafocal thermal necrosis under proper NIR laser illumination while sparing tissue-lacking particles and preventing damage to critical structures within and around the prostate, preserving baseline function. This paper details the course of AuroLase Therapy and a 1-year follow-up that a patient with localized PCa goes through. Post-therapy, a shutdown of the tissue microvasculature is expected at the treated area containing AuroShell Particles. Changes in the K^trans^ and ADC values assist in quantitatively evaluating tissue changes during the one-year follow-up. At 6 months and 1 year post-treatment, a successful AuroLase treatment was confirmed by negative targeted biopsies with no abnormalities observed on the mpMRI at the treated area. At the one-year follow-up, the treated area was degraded with a band-like scar on MR images. The clinical Phase I/II device trial is complete with one year of follow-up. The final report covering all of the patients will be published in the near future and the therapy is in the process of FDA clearance.

## 5. Materials and Methods

**Nanoparticle fabrication.** Oldenburg et al. [28,61] describe the dielectric core/metal shell nanoshell development and the rationale for their ability to produce a plasmon resonance tunable over the visible and near-infrared spectra. The optical properties of spherical particles are accounted for by Mie scattering theory, which was expanded to a particle consisting of a dielectric core with a metallic shell using the Drude model for metal permittivity. Nanospectra Biosciences, Inc. produces AuroShell^®^ Particles (nanoshells), composed of a 110–120 nm diameter silica core coated with a 10–15 nm thick gold shell and a layer of 6k molecular weight methoxypolyethylene glycol thiol (mPEG thiol) covalently bound to the surface, yielding a particle with a broad plasmon resonance centered at 780–800 nm (Figure 2Ai). The PEGylation assists in bypassing the reticuloendothelial system [43], mainly macrophages, increasing the nanoshells’ half-life circulation time. AuroShell Particles used for AuroLase^®^ Therapy are biochemically and metabolically inert and regulated as devices by the FDA, per ISO 10993 standards [62]. Formal GLP biocompatibility testing has complied with ISO-10993 and FDA G-95 guidelines. Numerous studies have been commissioned to verify a safety profile for AuroShell Particles in animal models [25,34] and as demonstrated in human trials [15] with no evidence of toxicity at a dose equal to or double that expected for clinical application.

**Laser delivery system.** The optical energy to activate the AuroShell Particles is delivered via the Laser Delivery Device (LDD, Nanospectra Biosciences, Inc., Houston, TX, USA). Supplementary Materials, Figure S1 shows the components of the LDD and related equipment, consisting of (1) the Laser Catheter Assembly—a dual lumen transparent catheter that both encloses the optical fiber and a cooling jacket to regulate temperature; (2) the Optical Fiber Diffuser (OFD)—an optical fiber terminated with a 1.8 cm long isotropic diffuser; (3) the Coolant Supply Set, which supplies sterile saline or water to the Laser Catheter Assembly; and (4) the Coolant Recovery Bag, which stores the coolant after a single pass through the laser catheter. Also shown is a pair of the wavelength-specific Laser Safety Eyewear, the integrating sphere power meter used to calibrate and confirm the laser output, and the custom AuroLase Laser Introducer—a 14-gauge catheter with a trocar/cannula (ALI, Nanospectra Biosciences, Inc., Houston, TX, USA) used to create a tract for the Laser Catheter.

Optical energy was generated from a dual-output, 9W, 810 nm diode laser (model EXPi, LiteCure/DJO/Enovis, Lewisville, TX, USA). A peristaltic pump (BT100-1L-B, Langer Instruments, Tucson, AZ, USA) circulated saline coolant through the dual-lumen Laser Catheter Assembly to regulate the development of the thermal coagulum. The average laser power used for the output dosimetry confirmation was calibrated using an integrating sphere optometer (P9710-2/upk-30, Gigahertz-Optik GmBH., Puchheim, Germany).

**Laser dose.** The laser dose has been chosen to realize an Arrhenius-type thermal ablation obtained during three minutes of laser illumination in the presence of nanoshells, but to be sub-ablative in normal tissue where endogenous absorbers less strongly absorb the near-infrared wavelength. This substantially confines photothermal ablation to neoplastic tissue and leads to a coagulo–necrotic region that conforms to the neoplastic tissue rather than to the location and shape of the laser applicator. As the therapeutic mechanism of action does not rely on tissue-specific molecular markers for accumulation or therapy, AuroLase Therapy is readily applicable for treating various solid tumors. The maximum sub-ablative laser dose in healthy tissue is a function of the optical properties (absorption and scatter) and blood perfusion, which vary by tissue and is designed to result in mildly hyperthermic temperatures of ~45–48 °C with a <5% chance of individual cell death [63]. The presence of AuroShell^®^ Particles combined with the same laser dose adds ~5–7 °C due to absorbed photon flux by the nanoparticles converting photon energy into vibrational energy, raising the temperature to 53–55 °C with 100% lethality as described by the Arrhenius process [16,64]. In an animal model, 3 min of 810 nm laser illumination at a laser power of 3 W with a 1.0 cm-long OFD was sufficient to generate ~ 4 mm radius of extended photothermal tissue damage around the optical fiber with sub-millimeter uncertainty in tissue-containing nanoshells [33]. Agarose tissue phantom models consisting of 0.7 optical density (OD) of nanoshells, within the range of concentration expected at the tumor site, were illuminated for 3 min at 6 W with a standard 1.8 cm long OFD coupled with an 810 nm laser; as a result, a homogeneous spheroidal treatment volume of 1.68 cm^3^ (2.05 cm in length and 1.25 cm in diameter) was achieved, reaching ΔT = 17 °C sufficient to cause in vivo tissue necrosis (Supplementary Materials, Figure S2). Based on these preclinical studies and the clinical trial [15], AuroShell^®^ Particles are expected to induce tissue damage that extends ~4 mm in radius under 3 min of continuous illumination at 5.7 W with an 810 nm laser coupled from a 1.8 cm long OFD (5.9 W/cm^2^ laser density at the fiber surface) while sparing healthy tissues that lack nanoparticles. These illumination conditions are 10 folds below the damage threshold (>7.9 × 10^10^ W/cm^2^) for nanoshells, as reported by Park et al. [65]. Thus, nanoshells are expected to maintain their shell–core structure post-laser-treatment under the reported clinical conditions.

**Particle-directed photothermal coagulation.** AuroLase Therapy shares features of both Photodynamic Therapy (PDT) and Laser Interstitial Thermal Therapy (LITT). Operationally, AuroLase Therapy resembles PDT with its exogenous, tumor-specific absorber, though using a laser irradiance ~10× higher than PDT, though only ~1/3 to 1/2 that of conventional LITT, to generate photocoagulative rather than photochemical response. The laser dose emitted from an isotropic diffuser is typically 4.3–8.3 W/cm^2^ at the fiber surface. The laser procedure is functionally distinct from, but analogizes to, brachytherapy, in that multiple parallel optical fiber insertions are required to treat a large volume by treating discrete small volumes (0.6–2.4 cm^3^) (Supplementary Materials, Figure S2). The objective is to use a nominally sub-ablative laser dose to generate coagulative necrosis specific to the targeted tumor that contains nanoshells while sparing nanoshell-free tissues. Clinical data revealed that the concentration of nanoshells in tumors is spatially heterogeneous and tends to be between 0.08 × 10^9^ to 2.3 × 10^9^ nanoshells/mL, causing sufficient thermal damage under a sub-ablative laser dose [15].

**Multiparametric MRI system.** The mpMRI scans were acquired with a Philips 3.0 Tesla MR scanner (Philips Medical System, Best, Netherlands). The primary sequences were anatomical images (axial and coronal *T*_2_w MRI) and functional MR images (axial DWI-EP sequence with b-values of 50 s/mm^2^ and 800 s/mm^2^, and axial DCE-MRI-*T*_1_w GR sequence). The same imaging protocols with a slide thickness of 3.0 mm (no gaps) were acquired at a different time point (screening and follow-ups) of the AuroLase Therapy. Sequence parameters were adjusted according to the tissue changes post-treatment (Supplementary Materials, Table S1). Prostate Imaging–Reporting and Data System version 2.1 (PI-RADS v2.1) was used for PCa scoring.

**Two-Day AuroLase**^®^ **Therapy.** A maximum of 120 days was allowed to elapse between the screening mpMRI and the nanoshell infusion for the planned treatment volume to be valid for laser targeting. The AuroLase Therapy procedure began with the AuroShell Particles infusion, followed a day later by the laser procedure.

**i.**  **Selection of Participants.**

The patient in this report participated in the clinical study according to the inclusion and exclusion criteria found on clinicaltrials.gov/study/NCT04240639 (accessed on 21 July 2024).

**ii.**  **Day 1—AuroShell^®^ Particles Infusion.**

On Day 1, seven pouches of 100 OD AuroShell Particles suspension were removed from the storage refrigerator and brought to room temperature. A total of 669 mL was infused into an 81.3 kg patient, yielding a dose of 8.2 mL/kg, ~10% over the planned dosing level of 7.5 mL/kg/dose, likely due to emptying the contents of the entire pouches of the product instead of using the infusion pump to regulate the total amount infused. Even so, the patient experienced no adverse reactions to the particle infusion and was released following the nanoparticles’ infusion.

**iii.**  **Day 2—Laser Treatment. *Patient preparation*.**

The laser procedure is performed under general anesthesia as the accurate co-registration of the mpMRI-generated model to the real-time ultrasound requires the patient to remain motionless as the optical fibers are positioned. Patients are intubated while positioned in the dorsal lithotomy position. A periprostatic nerve block is performed using 1% lidocaine without epinephrine via the perineum under transrectal ultrasound (TRUS) guidance. Rocuronium is used to prevent patient movement, which would degrade the precision of ultrasound mapping.

***MR imaging/ultrasound fusion transperineal guided therapy.*** MRI/US fusion biopsy with a transperineal approach is superior to a transrectal approach in detecting and sampling clinically significant PCa within an MRI-visible index lesion [66]. This could be a result of performing targeting through a stabilized template, and the needle is less likely to deflect during the transperineal approach. Thus, accurate placement of the Laser Catheter Assemblies in proximity to the target tumor was accomplished by first passing a 14-gauge trocar catheter (AuroLase Laser Introducer, Nanospectra Biosciences, Inc., Houston, TX, USA) through a disposable template grid (610-977, CIVCO, Coralville, IA, USA) secured to a stepper stage on which the TRUS probe was mounted. The relative position and orientation of the introducers, grid, and TRUS were tracked by a UroNav MR/US Fusion guidance system (UroNav, A Philips Healthcare System, Best, The Netherlands). Supplementary Materials Figure S7 is an example of the UroNav program screenshot taken during one of the 102 PCa laser treatments. While ultrasound imaging provides a live prostate scan, pre-MRI data were used to show intra-prostatic anatomical details and precisely locate the tumor volume relative to the prostate volume. The MR/US fusion images were performed by fitting the mesh or surface rendering obtained with the MRI with that of the live ultrasound scan. Optimum co-registration of these two imaging modalities requires (1) slow, steady acquisition of the ultrasound scan and (2) inclusion of the anterior fibro-muscular stroma in the prostate gland segmentation in the MR images as it cannot be differentiated from the prostate gland by ultrasound. Failure to meet these requirements decreases the value of MRI data and treatment resolution.

Furthermore, *T*_2_w MRI underestimates PCa size [45,46] by an average of 11 mm in tumor length and three folds in tumor volume when compared to whole-mount histopathology after resection, with the least accurate measurement in the longitudinal plane (base-apex axis) [45]. Thus, larger ablation margins were planned, covering the mpMRI-detected lesion and its surroundings (~7 mm minimum margin) in the transverse and longitudinal planes. Post MR/US co-registration, UroNav enabled 3-axis visualization of the introducers and, subsequently, the laser catheters relative to the transperineal grid, displaying the designed focal ablation volume (green) within the prostate gland (red), targeting the tumor area and its surroundings (Supplementary Materials, Figure S7).

***Laser treatment and conditions.*** The laser treatments for this case began 22.7 h after the completion of the nanoshell infusion. This permitted the nanoshells, which have a 3–6 h circulating half-life in the bloodstream to reach their maximum uptake in the tumor peri-vasculature space via the EPR effect [25,34]. Overall, the laser treatments occupied 41 min of a much longer procedure involving setting up the ultrasound visualization system, linking it with the UroNav fusion software version 3.0, and performing the treatment planning in real time.

***Laser introducer placement sequence.*** Introducer placement was planned based on the limited penetration of photons into prostate tissue, the conformation of the target ablation zone, and the interference posed by various anatomical features, e.g., the pubic bone, urethra, prostate capsule boundary, bladder, and critically, the proximity of the anterior rectal wall. For this, the fixed 3D relationship of the TRUS, template grid, and various anatomical features within the field surveilled by the UroNav was used to derive grid coordinates into which to insert the introducers. A total of seven 14-gauge catheter/trocars were inserted percutaneously within and around the tumor while avoiding the neurovascular bundle and urethra. The introducers’ placement sequence is shown in Supplementary Materials, Figure S3. The initial introducers effectively fix the prostate in position so that subsequent laser catheter placements and thermally induced edema would not distort the target region.

***Laser treatment sequence.*** Following AuroLase laser introducer placement, trocars were removed sequentially and replaced by the Laser Catheter Assemblies. Each of the two 810 nm lasers was coupled with a 1.8 cm long diffuser and placed sequentially in the targeted area. After their positions were confirmed with the ultrasound, they illuminated the region of interest for 3 min at 5.7 W with the sequence presented in Figure 3C, treating the anterior aspect first, then working toward the posterior aspect, i.e., toward the TRUS, as the accumulation of thermal damage tends to degrade the ultrasound image. Eleven laser treatments were performed along the seven tracts. Four of the treatments were carried out after retracting the laser catheter along their respective tracts to cover the targeted area in the longitudinal plane with 5 mm or 10 mm of pullback. According to this plan, the dimensions of the expected ablation zone were 2.0 cm × 2.0 cm (in-plane) and 2.8 cm (extent).

***Single laser illumination at the contralateral side of the prostate gland.*** As part of the study protocol, an additional single laser treatment was carried out at the same power setting and for the same 3 min duration outside the ablation region, ~24.7 mm lateral to the targeted tumor in the contralateral peripheral zone in the right side of the prostate gland (Supplementary Materials, Figure S4). The goal of this measurement was to verify that a single laser dosimetry was indeed sub-ablative.

While the laser portion of the prostate procedure is conducted under anesthesia, it is nonetheless minimally invasive as the percutaneous optical fiber applications do not require surgical closure but only temporary wound packing. Patients were discharged with only temporary activity level restrictions after recovering from anesthesia. Because of the optical fiber penetrations of the prostate, approximately two days of mild discomfort and hematuria are expected. Patients >65 years old and with a prostate volume larger than 42 cc have a higher risk of urinary retention post-transperineal-targeting [67]. Thus, patients are discharged with a urinary catheter following AuroLase Therapy.

**Treatment assessment:** Post-AuroLase Therapy, the following tests were performed to assess the treatment efficacy: PSA, mpMRI, biopsies, and self-assessments [55] at timepoints approved by the FDA. For purposes of the clinical trial, though not a part of the routine therapy, efficacy and acute ablation volume was assessed by contrast-enhanced MRI 48–96 h after laser illumination to allow time for the appearance of coagulative necrosis, but prior to the reconfiguration of tissue by lytic action. The appearance of a ‘void’ space on the DCE-MRI (CA wash-in/wash-out) color map and on its K^trans^ (permeability) map would be more generally expected than lesion shrinkage for treatment evaluation. At one month post-therapy, a physical examination, PSA test, and personal assessments were completed. At 6 months, a series of laboratory tests and personal assessments were accompanied by mpMRI and targeted MR/US transperineal fusion biopsies in which 4 cores were collected from the treated region. At one year, the laboratory tests and personal assessments were repeated, accompanied by mpMRI scans of the abdomen and a systematic MR/US transperineal fusion biopsy in which 12 cores were collected per standard of care, targeting the treatment zone and covering the entire prostate gland.

A treatment was scored as successful if the following conditions were met: (1) the DCE color map and its K^trans^ on Day 4/6 showed a void space at the treated/lesion site, demonstrating an absence of perfusion representing a complete shutdown to tumor vasculature; (2) no mpMRI abnormalities such as hyperintense signals on DWI and early contrast enhancements on DCE-MRI in the originally treated zone were noted at 12 months post-therapy; and (3) there was negative directed biopsy for malignancy within the treated area at 12 months post-AuroLase Therapy [68].

Per standard of care, patients remained under surveillance for a couple of years post-treatment, but such follow-up was outside the scope of this study. Nevertheless, patients who experienced acute or chronic hepatic dysfunction as evidenced by clinically significant abnormalities in albumin, total protein, or prothrombin time, or evidence of hepatic injury with clinically significant (>grade 1) changes in AST, ALT, ALP, bilirubin, or GGT values were followed until resolution. Patients consented for up to five years to track their disease status and progression or recurrence, if any. Urinalysis and cultures were performed at baseline and at the 180-day and 360-day visits. All other labs were acquired per standard of care, including CBC, chem 7, creatinine, liver function, coagulation parameters, PSA, etc.

**Multiparametric MR imaging analysis:** Invivo DynaCAD software (version 4.0, Philips Medical System, Best, The Netherlands) was used to process the mpMRI data by performing prostatic segmentation and placing a region of interest (ROI) over a lesion, whole/core treated area, and reference (healthy tissue). The system used Prostate Imaging–Reporting and Data System (PI-RADS) version 2.1 to score the lesion [35]. The system was used to calculate high-b-value DWI from b-values of 50 s/mm^2^ and 800 s/mm^2^ and generate and analyze the ADC, DCE, and K^trans^ maps. The DCE color map is a composite color map based on K^trans^ (permeability) and V_e_ (extravascular extracellular space volume fraction), presenting three patterns of contrast agent signal enhancement curve type before, during, and after CA injection (70 *T*_1_w MR scans), presented with three color codes (blue, green, and red); blue is for a slow persistent signal intensity increase; green is for a fast signal enhancement followed by a plateau phase (<10% from the peak); red is for a rapid enhancement followed by a wash-out/decline phase [69].

**Clinical trial organization:** The specific case presented here is intended to illustrate the clinical development of AuroLase Therapy. It was one of a combined 102 patients involved in the device trial (ClinicalTrials.gov # NCT02680535 and # NCT04240639) designed as an open-label, multi-center, single-dose study of AuroLase Therapy used against low- to intermediate-risk PCa with Gleason score 6–7 (ISUP GGG ≤ 3). The study was confined to MRI-identified, biopsy-confirmed MR/US fusion diagnoses of prostate disease with one or two distinct lesions. After signing Informed Consent and confirming study eligibility, patients were enrolled.

## Figures and Tables

**Table 1:** PSA and self-assessments.

	Screening/Baseline	+20 Days	+182 Days	+373 Days
PSA (ng/mL)	4.57	6.63	1.68	2.16
Prostate volume (cc)	34.4	35.9	24.8	26.1
MR/US fusion transperineal biopsy (# of cores at the targeted area)	(5/6 cores) Prostatic adenocarcinoma GGG 1	-	(4/4 cores) Benign–negative for malignancy	(1/1 core) Benign–negative for malignancy
IPSS (0–35)	10	5	5	9
SHIM (0–25)	19	20	20	14

## Data Availability

The original contributions presented in the study are included in the article/Supplementary Material; further inquiries can be directed to the corresponding author/s.

## Supplementary Materials

The following supporting information can be downloaded at: https://www.mdpi.com/article/10.3390/nano14151261/s1, [15,16,70] is cited in the Supplementary Materials file. **Figure S1**. The Laser Delivery Device (LDD). **Figure S2.** The distal tip of the Laser Catheter Assembly (LCA) with the ablation zone generated from an Optical Fiber (OPF) with a 1.8 cm Optical Fiber Diffuser (OFD) end. **Figure S3**. Treatment planning introducer insertion sequence. **Figure S4**. The localization of the single laser treatment site [control burn] relative to the urethra and the treated tumor on T2w MR images performed three days post-AuroLase® Therapy (Day 5). **Figure S5**. Contrast agent (CA) signal enhancement at the tumor site (left anterior peripheral zone-PZa) versus healthy tissues (right PZa) at screening. **Figure S6**. A summary of the relevant multiparametric MR images at screening (≥ Day -120) and follow-up (Day 5, Day 181, and Day 374) post-AuroLase® Therapy. **Figure S7.** An example of the use of the UroNav system during an AuroLase® Therapy laser treatment under live MR/US fusion guidance. **Table S1**. Multiparametric MRI parameters at screening and follow up under 3T MRI scanner.
