# Supplementary material for: A Detailed Clinical Case of Localized Prostate Tumors Treated with Nanoparticle-Assisted Sub-Ablative Laser Ablation
Source: Nanomaterials (Basel). 2024 Jul 28;14(15):1261. doi: 10.3390/nano14151261 (PMC11313886; doi:10.3390/nano14151261)
Supplement: Supplementary file 1 [file nanomaterials-14-01261-s001.zip › nanomaterials-3088979-supplementary.pdf]

Supplementary Information for

A Detailed Clinical Case of Localized Prostate Tumors Treated with Nanoparticle-assisted Sub-ablative Laser Ablation

Yara Kadria-Vili [1], Jon A. Schwartz [1], Thomas J. Polascik [2], Glenn P. Goodrich [1], David Jorden [1], Diane Pinder [2], Naomi J. Halas [3-6], and Ardeshir R. Rastinehad [7,*]

[1] Nanospectra Biosciences, Inc., Houston, TX 77054, USA

[2] Department of Urology, Duke University Medical Center, Durham, NC 27710, USA

[3] Laboratory for Nanophotonics, Rice University, 6100 Main Street, Houston, TX 77005, USA

[4] Department of Chemistry, Rice University, 6100 Main Street, Houston, TX 77005, USA

[5] Department of Electrical and Computer Engineering, Rice University, 6100 Main Street, Houston, TX 77005, USA

[6] Department of Physics and Astronomy, Rice University, 6100 Main Street, Houston, TX 77005, USA

[7] Smith Institute for Urology at Lenox Hill Hospital, Northwell Health, Zucker School of Medicine at Hofstra/Northwell, New York, NY 10075, USA

* Correspondence: arastine@northwell.edu*Authors to whom correspondence should be addressed

This PDF file includes the following:

Figures. S1 to S7
Elemental Analysis
Multiparametric Magnetic Resonance Imaging (mpMRI) parameters at screening and follow up (Table S1).

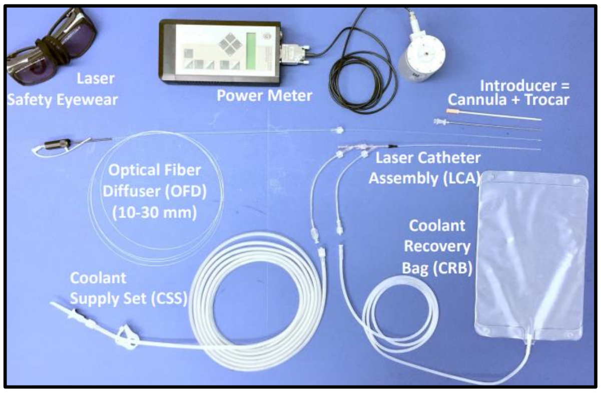

Figure S1: The Laser Delivery Device (LDD). The LDD consists of the Laser Catheter Assembly (LCA), Optical Fiber Diffuser (OFD), Coolant Supply Set (CSS), and Coolant Recovery Bag (CRB). Also shown are a representative AuroLase Laser Introducer (a 14-gauge trocar/cannula catheter), a pair of 810 nm Laser Safety Eyewear, and an integrating sphere optical power meter.

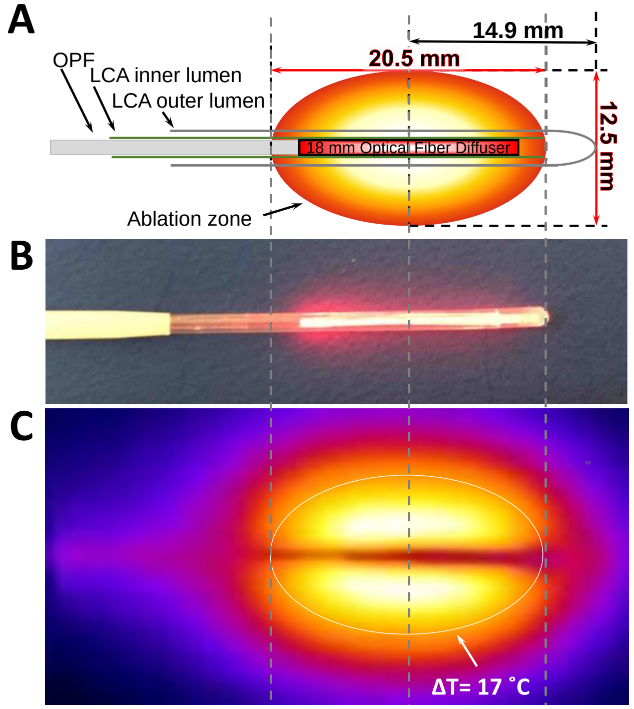

Figure S2: The distal tip of the Laser Catheter Assembly (LCA) with the ablation zone generated from an Optical Fiber (OPF) with a 1.8 cm Optical Fiber Diffuser (OFD) end. (*A*) A schematic of the distal LCA (Fig. S1*B*) overlaid with the nominal ablation zone, highlighting the dual-lumen cooling channel and the ablation zone relative to the LCA tip. (*B*) The LCA tip enclosed 1.8 cm-long OFD within the dual lumen LCA. (*C*) A thermal image of the heat distribution in the plane of the LCA within a homogenous phantom consisting of 0.7 optical density (OD) AuroShell® Particles uniformly dispersed in the agarose plus hemoglobin medium under 810 nm laser exposure. After 3 minutes of illumination at 6 W, a ΔT of 17 ˚C extended to the marked boundary (solid white line), creating a 1.68 cm^3 oblate spheroidal ablation volume (20.5 mm long ×12.5 mm maximum diameter) centered 14.9 mm from the tip of the LCA.

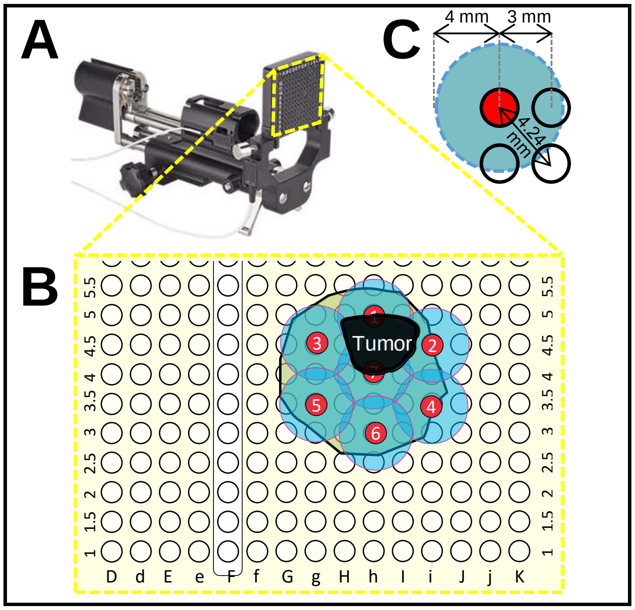

Figure S3: Treatment planning introducer insertion sequence. (*A*) A transperineal grid mounted to a stage that also secures the Transrectal Ultrasound (TRUS, not shown). (*B*) The planned ablation zone (black outline), covering the tumor (black) and the surrounding margin, superimposed on the transperineal grid. The numbered red circles represent the placement sequence of the seven trocars utilized for this treatment. Because the tumor abutted the capsule (*main text,* Fig. 3*C*), the margins were scant at the top right of the image (top left of the prostate gland), avoiding unnecessary photothermal damage to the nearby soft tissues and muscles. Thus, probes 1,2,3, and 7 covered the tumor and probes 4,5, and 6 covered the margin inferior to the tumor. (*C*) An 8 mm ablation zone diameter is expected after 3 minutes of illumination at 5.7 W @ 810 nm. The transperineal grid consists of numbered rows and lettered columns, with the 14-gauge holes laid out on a 3 mm center-to-center square grid.

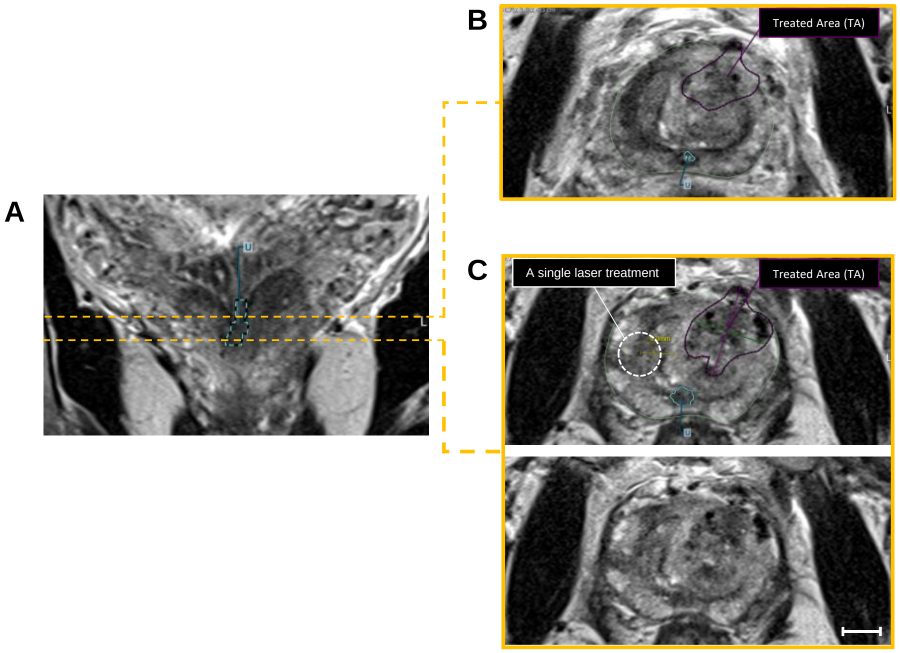

Figure S4: The localization of the single laser treatment site [control burn] relative to the urethra and the treated tumor on T_2w MR images performed three days post-AuroLase® Therapy (Day 5). (*A*) A coronal T_2w MR image and (*B*-*C*) the corresponding axial T_2w MR images along the dashed yellow lines with 6 mm spacing from their centers (slice thickness= 3 mm with no gap). The prostate capsule was denoted with a solid green line; the treatment at the tumor-free site, presented with a low T_2 signal intensity (dashed white circle= control burn), was found ~9.0 mm from the urethra (U, cyan) aligned with the treatment plan (Fig. S3). (Scale bar = 10 mm)

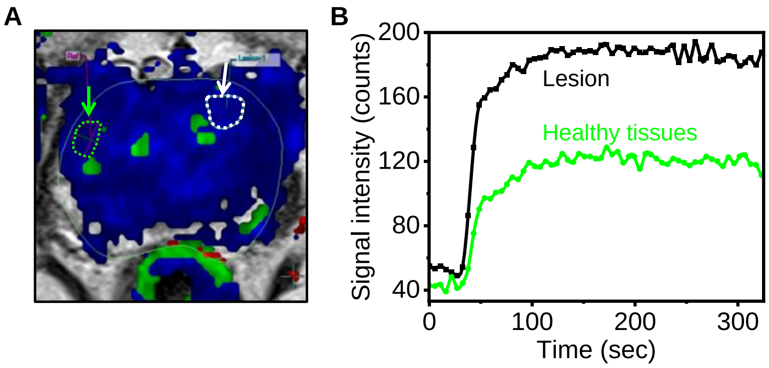

Figure S5: Contrast agent (CA) signal enhancement at the tumor site (left anterior peripheral zone-PZa) versus healthy tissues (right PZa) at screening. (*A*) DCE (CA wash-in/wash-out) overlaid on an axial T_2w MRI. (*B*) The dynamic contrast enhancement kinetics of the regions with tumor-free tissue (right PZa-ref-green arrow) and tumor (left PZa-white arrow) reveal the T_1w MRI signal enhancement before, during, and after the injection of the CA where the tumor (black) has a higher CA enhancement than healthy tissues (green).

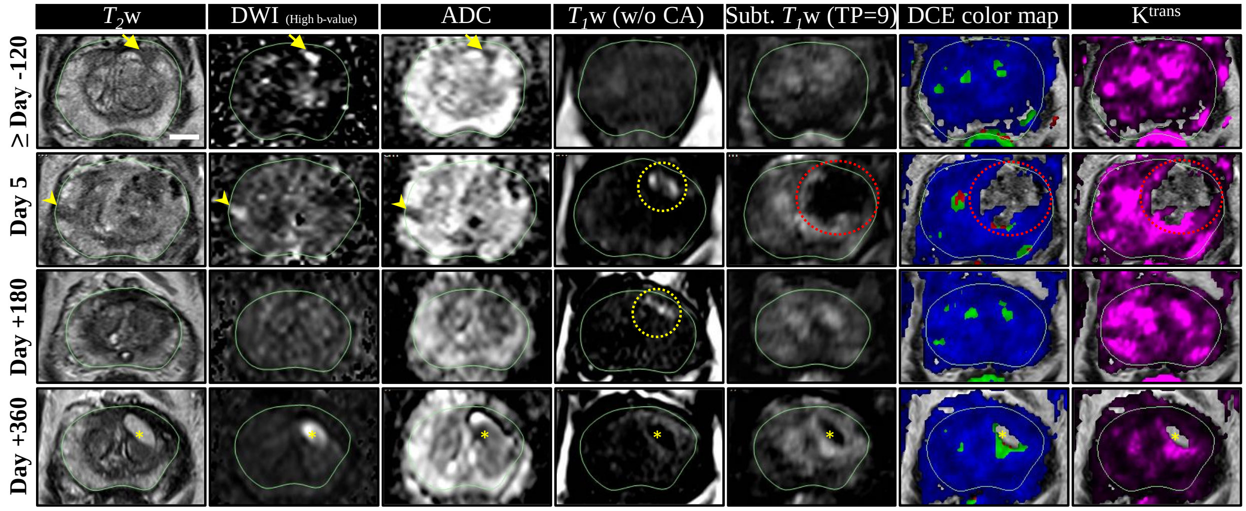

Figure S6: A summary of the relevant multiparametric MR images at screening (≥ Day -120) and follow-up (Day 5, Day 181, and Day 374) post-AuroLase® Therapy. The axial images from left to right are: T_2w MRI, high b-value Diffusion Weighted Image (DWI), Apparent Diffusion Coefficient (ADC) map, T_1w MRI (without background subtraction and before contrast agent (CA) injection), subtracted T_1w MRI AT timepoint = 9, DCE color map (CA wash-in/wash-out), and T_2w MRI with K^{trans} map overlay. Yellow arrow: tumor with PI-RADS 4 using the PI-RADS v2.1 scoring system, which was treated with AuroLase® Therapy. Dashed yellow circle: evidence of hemorrhage. Dashed red circle: control burn site with absent localized perfusion due to successful AuroLase® Therapy photothermal ablation covering the tumor site and its surroundings. Necrosis and fibrosis at the treated area on Day 5 post-treatment show heterogenous low T_2 signal intensities, low signal on DWI/ADC map, and lack of signal enhancement on DCE. At Day +360, a cystic change (*) appeared as high signal intensity on T_2w MRI, hyperintense on high b-value DWI, hypointense on ADC map, low intensity on T_1w MRI (w/o CA), and lack of enhancement on background subtracted T_1w MRI (Subt. T_1w) and DCE color map. An additional lesion (lesion # 2, arrow head) with PI-RADS 4 at the right posterolateral PZ midgland was evident on Day 5 mpMRI for the first time with low signal intensities on T_2w MRI, high on DWI, and low on ADC map. There was no sign of the 2nd lesion on Day 180 and Day +360 mpMRI scans. However, at one-year post-therapy, one biopsy confirmed positive for prostatic adenocarcinoma at the right transitional zone with a Gleason score 6 (Grade Group 1) and another confirmed atypical small acinar proliferation at the right anterior PZ. The mpMRI processing was performed using Invivo DynaCAD (version 4.0.0, Philips Medical System, Best, Netherlands) (Scale bar= 1 cm).

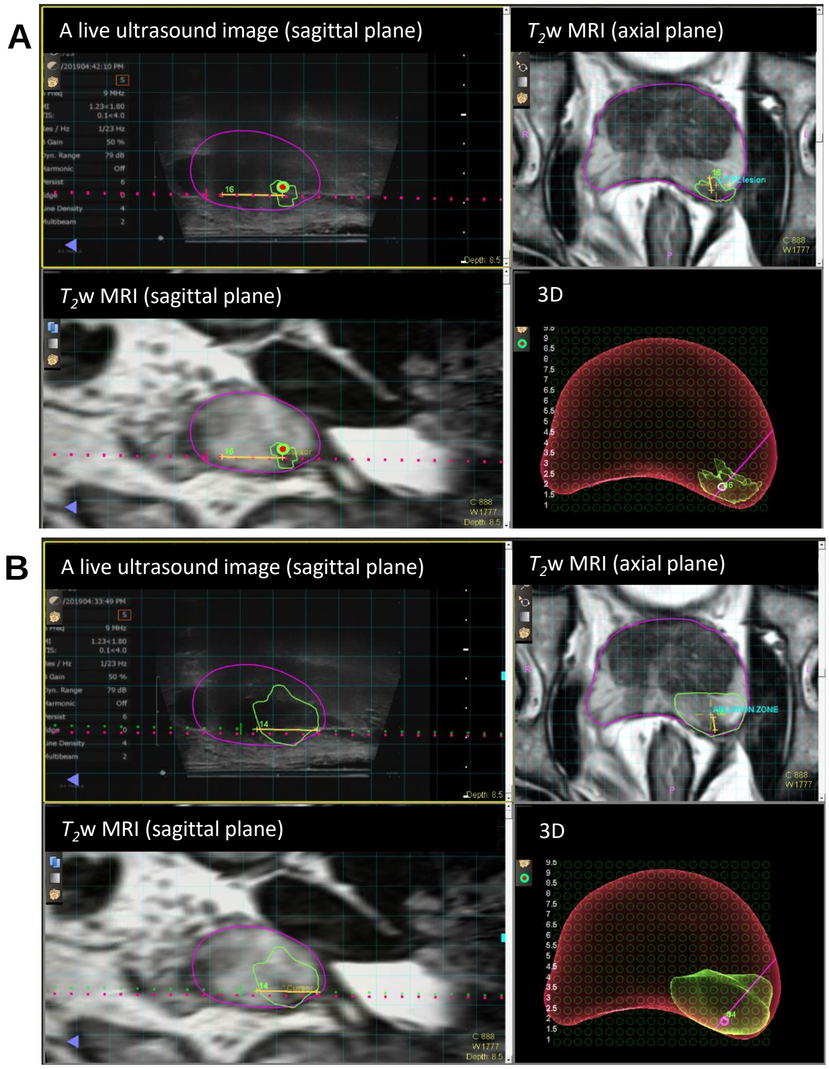

Figure S7: An example of the use of the UroNav system during an AuroLase® Therapy laser treatment under live MR/US fusion guidance. Note: this screen image is for a different PCa patient than the one discussed in the main text. This patient had a tumor in the left posterior PZ of the prostate. (*A*) *Top left*: a live ultrasound image in a sagittal plane; *top right/bottom left*: pre-treatment axial/sagittal T_2w MR images for prostate gland segmentation (purple) and targeted lesion visualization (green); *right bottom*: targeted lesion volume (green) relative to the transperineal grid (white) for trocars distribution within the targeted area. The horizontal dotted line on the top/bottom left US/MR images shows the trajectory of a trocar inserted into position I2 of the grid. The pink line is the axis of the ultrasound plane. While ultrasound provides live imaging guidance, MRI provides anatomical details, improving tumor visualization and targeting. The UroNav screenshot in (*B*) highlighted the targeted ablation volume (the lesion plus a margin) in green.

Elemental analysis. Measurements were conducted on targeted MR/US fusion transperineal biopsies, confirming the presence of AuroShell® Particles within the tumor (2.62 µg Au/g of wet tissue), which is within the reported range of concentration of 1.15-33.12 µg Au/g ((0.78-22.60) $\times 10^8$ nanoshell/mL) found within the PCa tumor of the fourteen PCa patients treated with AuroLase® Therapy [1].

An additional sample was collected from the right side of the prostate gland, where a single laser treatment was performed, revealing the presence of AuroShell® Particles (3.16 µg Au/g of wet tissue) with an equal order of magnitude found in the tumor. This could be due to the collection of tissues from the nearby tumor (lesion # 2) at the right posterolateral PZ midgland, which was MRI visible on Day 5 for the first time (*SI Appendix* Fig. S6), or a Benign Prostatic Hyperplasia (BPH) nodule in the transition zone close to the PZ boundary. The initial safety evaluation study of AuroShell® Particles in 22 PCa patients found no significant difference between gold content in PCa and BPH [2] (unpublished data). BPH was found to be low in adherens junction protein (E-cadherin) compared to healthy prostate tissue. E-cadherin plays a significant role in tight junction development and maintenance. A low E-cadherin increases the epithelium barrier permeability in BPH [3]. Thus, during the prostate treatment with AuroLase® Therapy, the accumulation of the AuroShell® Particles should be expected in the BPH and tumor due to the BPH's dysfunction barriers and the enhanced permeability and retention effect, respectively. Nevertheless, normal contrast enhancement DCE MRI and kinetic profile were detected after a single laser treatment at this site (*main text*, Fig. 5*H*), similar to the enhancement-kinetic profile for healthy tissues (*SI Appendix*, Fig. S5), suggesting no significant evidence of necrotic tissue. These findings indicate that the applied laser illumination condition was safe under a single laser illumination, causing insufficient tissue damage.

Multiparametric MRI parameters at screening and follow up.

The mpMRI scans were acquired with a Philips 3.0 Tesla MR scanner (Philips Medical System, Best, the Netherlands). The same imaging protocols with a slide thickness of 3.0 mm (no gaps) were acquired at a different time point (screening and follow-ups) of the AuroLase® Therapy. Table S1 summarizes the primary sequences (axial T_2w MRI, DWI at various b-values, and DCE MRI) and their parameters.

Table S1: Multiparametric MRI parameters at screening and follow up under 3T MRI scanner.

<table>
<tr><th></th><th></th><th colspan="3">Post-AuroLase® Therapy</th></tr>
<tr><th>Sequence</th><th>Screening</th><th>Day 5</th><th>Day 180</th><th>Day +360</th></tr>
<tr><td colspan="5">Axial T_2w MRI (SE)</td></tr>
<tr><td>FOV (cm^2)</td><td>16×16</td><td>16×16</td><td>18×18</td><td>16.3×18</td></tr>
<tr><td>Slice thickness/gap (mm)</td><td colspan="4">3/0</td></tr>
<tr><td>Acquisition matrix</td><td colspan="2">384×384</td><td>640×640</td><td>580×640</td></tr>
<tr><td>TE (ms)</td><td colspan="2">96.0</td><td>124</td><td>134</td></tr>
<tr><td>TR (ms)</td><td colspan="2">4,100</td><td>3,500</td><td>2,880</td></tr>
<tr><td>FA (˚)</td><td>120</td><td>147</td><td>140</td><td>160</td></tr>
<tr><td>Avg</td><td colspan="2">4</td><td colspan="2">3</td></tr>
<tr><td colspan="5">Axial DWI (SS-EP)</td></tr>
<tr><td>FOV (cm^2)</td><td>14.7×7.6</td><td>16×9.7</td><td>18×18</td><td>14.3×18</td></tr>
<tr><td>Slice thickness/gap (mm)</td><td colspan="4">3/0</td></tr>
<tr><td>Acquisition matrix</td><td>116×60</td><td>116×70</td><td>232×232</td><td>184×232</td></tr>
<tr><td>TE (ms)</td><td>69.0</td><td>75.0</td><td>68.0</td><td>90.0</td></tr>
<tr><td>TR (ms)</td><td colspan="2">6,300</td><td>5,700</td><td>6,400</td></tr>
<tr><td>FA (˚)</td><td colspan="4">90.0</td></tr>
<tr><td>b-values (sec/mm^2)</td><td colspan="4">50, 800</td></tr>
<tr><td>Avg</td><td colspan="2">8</td><td colspan="2">11</td></tr>
<tr><td colspan="5">Axial DCE (T_1w GRE imaging)</td></tr>
<tr><td>FOV (cm^2)</td><td colspan="4">22×22</td></tr>
<tr><td>Slice thickness (mm)</td><td colspan="4">3</td></tr>
<tr><td>Acquisition matrix</td><td colspan="4">192×192</td></tr>
<tr><td>TE (ms)</td><td colspan="4">1.44</td></tr>
<tr><td>TR (ms)</td><td colspan="4">4.09</td></tr>
<tr><td>FA (˚)</td><td colspan="4">15.0</td></tr>
<tr><td>Avg</td><td colspan="4">1</td></tr>
<tr><td>Timepoints / time (min:s) for acquisition</td><td colspan="4">70 / 6:12</td></tr>
</table>

Note- SE= spin-echo sequence, DWI= Diffusion Weighted Image, SS-EP= single-shot echo-planar sequence, DCE- Dynamic Contrast-Enhanced, GRE= gradient-recalled echo, FOV=field of view, TE= echo time, TR= repetition time, FA= flip angle, Avg= number of signal averages, EP= a single shot echo-planar pulse sequence

References

(1) Rastinehad, A. R.;Anastos, H.;Wajswol, E.;Winoker, J. S.;Sfakianos, J. P.;Doppalapudi, S. K.;Carrick, M. R.;Knauer, C. J.;Taouli, B.;Lewis, S. C.;Tewari, A. K.;Schwartz, J. A.;Canfield, S. E.;George, A. K.;West, J. L.;Halas, N. J. Gold nanoshell-localized photothermal ablation of prostate tumors in a clinical pilot device study. *PNAS* **2019**, *116*, 18590-18596.

(2) Stern, J. M.;Solomonov, V. V. K.;Sazykina, E.;Schwartz, J. A.;Gad, S. C.;Goodrich, G. P. Initial evaluation of the safety of nanoshell-directed photothermal therapy in the treatment of prostate disease. *Int. J. Toxicol.* **2016**, *35*, 38-46.

(3) Li, F.;Pascal, L. E. E-cadherin is downregulated in benign prostatic hyperplasia and required for tight junction formation and permeability barrier in the prostatic epithelial cell monolayer. *Prostate* **2019**, *79*, 1226-1237.
